# Mutant p53-reactivating compound APR-246 synergizes with asparaginase in inducing growth suppression in acute lymphoblastic leukemia cells

**DOI:** 10.1038/s41419-021-03988-y

**Published:** 2021-07-15

**Authors:** Sophia Ceder, Sofi E. Eriksson, Ying Yu Liang, Emarndeena H. Cheteh, Si Min Zhang, Kenji M. Fujihara, Julie Bianchi, Vladimir J. N. Bykov, Lars Abrahmsen, Nicholas J. Clemons, Pär Nordlund, Sean G. Rudd, Klas G. Wiman

**Affiliations:** 1 Department of Oncology-Pathology, Karolinska Institutet, BioClinicum J6, Stockholm, Sweden; 2 Institute of Molecular and Cellular Biology, A*STAR, Singapore, Singapore; 3 Science For Life Laboratory, Department of Oncology-Pathology, Karolinska Institutet, Solna, Sweden; 4 Cancer Research Division, Peter MacCallum Cancer Centre, Melbourne, VIC Australia; 5 Sir Peter MacCallum Department of Oncology, The University of Melbourne, Parkville, VIC Australia; 6 Aprea Therapeutics AB, Solna, Sweden

**Keywords:** Cancer therapy, Haematological cancer

## Abstract

Asparaginase depletes extracellular asparagine in the blood and is an important treatment for acute lymphoblastic leukemia (ALL) due to asparagine auxotrophy of ALL blasts. Unfortunately, resistance occurs and has been linked to expression of the enzyme asparagine synthetase (ASNS), which generates asparagine from intracellular sources. Although *TP53* is the most frequently mutated gene in cancer overall, *TP53* mutations are rare in ALL. However, *TP53* mutation is associated with poor therapy response and occurs at higher frequency in relapsed ALL. The mutant p53-reactivating compound APR-246 (Eprenetapopt/PRIMA-1Met) is currently being tested in phase II and III clinical trials in several hematological malignancies with mutant *TP53*. Here we present CEllular Thermal Shift Assay (CETSA) data indicating that ASNS is a direct or indirect target of APR-246 via the active product methylene quinuclidinone (MQ). Furthermore, combination treatment with asparaginase and APR-246 resulted in synergistic growth suppression in ALL cell lines. Our results thus suggest a potential novel treatment strategy for ALL.

## Introduction

*TP53* is the most frequently mutated gene in cancer [1]. The most common type of *TP53* mutations is missense mutation that results in expression of functionally deficient p53 protein that fails to bind DNA and transactivate p53 target genes. Mutant p53 can exert a dominant-negative effect on wild-type p53, as recently demonstrated in myeloid malignancies [2], and may in some cases display so called gain-of-function activities that promote tumor development in various ways [3–5]. The mutant p53-targeting compound APR-246 (Eprenetapopt/PRIMA-1Met) is currently being tested in phase III clinical trials in myelodysplastic syndrome (MDS) and in several phase II trials in other hematological malignancies harboring *TP53* mutations and has recently received Food and Drug Administration (FDA) Fast Track Designation for mutant *TP53* acute myeloid leukemia (AML) and MDS. Both APR-246 and its structural analog PRIMA-1 are spontaneously converted to methylene quinuclidinone (MQ), a thiol-reactive Michael acceptor that binds covalently to cysteines in the p53 core domain [6]. APR-246 (through MQ) reactivates mutant p53 and induces tumor cell death [6, 7]. MQ conjugates antioxidant glutathione (GSH) [6, 8] and also targets redox-sensitive proteins of the cellular antioxidant defense systems, such as thioredoxin reductase (TrxR1) [9], thioredoxin, glutaredoxin, and ribonucleotide reductase [10]. Furthermore, the reversible binding of MQ to GSH contributes to an intracellular pool of active drug [11]. Thus, given the documented polypharmacology of APR-246/MQ, it is conceivable that this compound targets other proteins and pathways in cancer cells.

For the past four decades, asparaginase (ASNase) has been an important therapeutic agent for acute lymphoblastic leukemia (ALL) [12]. Despite its long clinical use, the exact molecular mechanism of action of ASNase in ALL is not fully understood [12, 13]. It is thought that ASNase depletes asparagine in the bloodstream, and while normal cells can synthesize asparagine intracellularly via the enzyme asparagine synthetase (ASNS), ALL cells have defective ASNS expression and thus rely on the uptake of extracellular asparagine from the blood [12–15]. It has also been proposed that the glutaminase activity of ASNase is important for its mechanism of action and contributes to its antitumor activities [12, 16, 17], since cancer cells are often dependent on glutamine [18]. Development of resistance to ASNase is an important clinical problem [12] and for a long time such resistance has been associated with ASNS expression [15, 19–22]. However, it remains unclear whether ASNS expression can predict ASNase response [23]. *TP53* mutations are rare in ALL in general but occur more frequently in relapsed ALL and are strongly associated with poor therapy response and poor prognosis [4, 24, 25].

CEllular Thermal Shift Assay (CETSA) is a biophysical method that was initially developed to study in situ drug–target engagement from lysate, cells, or tissues [26–28]. Upon heat treatment, proteins denature and precipitate. However, if the binding of a compound, or protein–protein, protein–RNA, or protein–DNA interactions, lead to an increased melting temperature of a protein, i.e., thermal stabilization, the protein will remain detectable in the soluble fraction. The combination of CETSA with mass spectrometry (MS-CETSA) proved to be a powerful tool to study direct and indirect cellular effects on a proteome-wide scale such as formation of interactions during various states of the cell cycle, changes in redox biology, or effects of posttranslational modifications [29–31].

We have performed MS-CETSA following treatment of cells with the APR-246 active product MQ, to identify protein targets that may offer novel therapeutic strategies. This identified ASNS as one of the top five proteins that were thermally stabilized following MQ treatment, suggesting that MQ directly interacts with or indirectly affects ASNS. Given the possible role of ASNS in ASNase drug resistance in ALL and the previously demonstrated efficacy of APR-246 in ALL mouse models [32], we also evaluated combination treatment of APR-246 and ASNase. We observed synergistic growth suppression in a panel of ALL cell lines, thus suggesting that APR-246 in the combination with ASNase may be a promising novel therapeutic strategy in ALL.

## Results

### MS-CETSA identifies ASNS as a putative MQ target

**Fig. 1:**
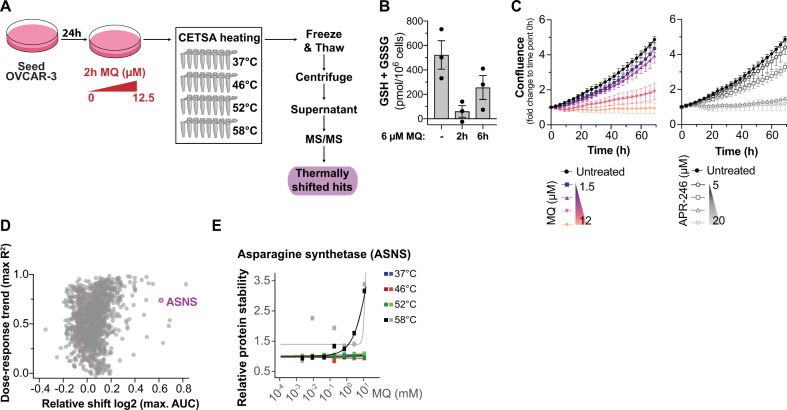
MS-CETSA identifies asparagine synthetase (ASNS) as a putative MQ target. **A** Overview of experimental set-up for MQ treatment of OVCAR-3 cells and MS-CETSA 2 h after treatment. **B** Total glutathione (GSH + GSSG) measured by enzymatic recycling assay in OVCAR-3 cells after 2 or 6 h of 6 µM MQ treatment. Untreated control was harvested at the 6 h time point. *n* = 3. **C** Confluence of OVCAR-3 cells at the indicated MQ and APR-246 concentrations as assessed by Incucyte. Gradients indicate treatment concentrations of 1.5, 3, 6, and 12 µM MQ and 5, 7.5, 10, and 15 µM APR-246, respectively. *n* = 2–3 (except for 15 μM where is *n* = 1). **D** Dot plot of the dose–response trend and relative shift of all thermally stabilized hits by MS-CETSA after MQ treatment in OVCAR-3 cells as shown in **A**. ASNS is indicated in purple. **E** MS-CETSA thermal shift response of ASNS with increasing concentrations of MQ at the indicated temperature in OVCAR-3 after 2 h of treatment. Different colors of the same temperature indicate separate MS runs. Relative protein stability is compared to vehicle control. MQ concentrations used were 0.0025, 0.01, 0.05, 0.2, 0.8, 3.1, and 12.5 µM.

Since APR-246 is converted to the active product MQ, a Michael acceptor that reacts reversibly with cellular thiols [6, 11], it is plausible that APR-246 targets a number of proteins other than what is known to date. Thus, we used MS-CETSA to identify novel proteins affected by MQ/APR-246. We selected the ovarian cancer cell line OVCAR-3, which carries the well-characterized *TP53* hot spot missense mutation R248Q, as an appropriate cell model for these studies. We have previously reported that this cell line responds to APR-246 treatment [11], which we document in further detail here (see below). We treated OVCAR-3 (*TP53* R248Q) with a range of concentrations of MQ for 2 h (discussed further below), after which cells were harvested and heated at 37, 46, 52, and 58 °C to denature and precipitate unfolded proteins (Fig. 1A). After cell lysis and removal of denatured/precipitated proteins by centrifugation, the supernatant was analyzed by MS/MS (Table S1). Proteins that were thermally stabilized or destabilized upon MQ treatment were considered potential hits that were either targeted by covalent MQ binding or indirectly affected via, for example, posttranslational modifications, redox modification, protein–protein interactions or binding of cellular metabolites, such as nucleotides or amino acids [29, 30, 33, 34].

While treatment with up to 12.5 µM of MQ for 2 h did not affect cell viability (Fig. S1A), it drastically depleted total glutathione (GSH + GSSG; Fig. 1B). However, after 6 h the total glutathione levels increased, indicating that the cells had begun to replenish their antioxidant reservoir. Similarly, increased xCT expression was observed after 2 h of MQ treatment but was not sustained at the 24-h time point (Fig. S1B). Antiporter xCT is regulated by master antioxidant sensor nuclear factor erythroid 2-related factor 2 (NRF2) and provides glutathione building blocks by importing cystine [35, 36]. MQ only showed inhibition of recombinant glutathione reductase (GR) activity at 100-fold higher concentrations than the concentrations used (Fig. S1C) and is therefore unlikely to affect the measurement in the GR recycling assay. Although cell viability was not affected at the harvesting time point with treatment up to 12.5 µM MQ (Fig. S1A), cell death was observed at later time points (Fig. S1D), as also shown by cell confluency (Fig. 1C) and caspase-3 cleavage (Fig. S1E). MQ was more potent than its prodrug APR-246 at the same concentrations (Fig. 1C) and induced caspase-3 cleavage at an earlier time point (Fig. S1E) as expected, since conversion of APR-246 to MQ takes certain time. Taken together, these data demonstrate that the concentrations of MQ chosen for the MS-CETSA study were sufficient to elicit anticancer phenotypes associated with APR-246 treatment and should therefore allow identification of biologically relevant targets.

ASNS, an enzyme that synthesizes asparagine from aspartate (Fig. S1F), was among the top five thermostabilized hits (Fig. 1D) and showed strong thermal shift at 58 °C (Fig. 1E). Expression of ASNS has been implicated in resistance to the important anti-leukemic therapy ASNase [15, 19–22], which has been in clinical use for decades to treat ALL, and thus we selected the hit ASNS for follow-up studies given the potential clinical relevance in using APR-246 as a novel strategy to overcome ASNase resistance in this disease. In summary, our MS-CETSA study identified ASNS as a thermally stabilized protein upon treatment with APR-246 active product MQ, suggesting that MQ may directly or indirectly alter ASNS activity.

### MQ thermostabilizes ASNS in ALL cells

**Fig. 2:**
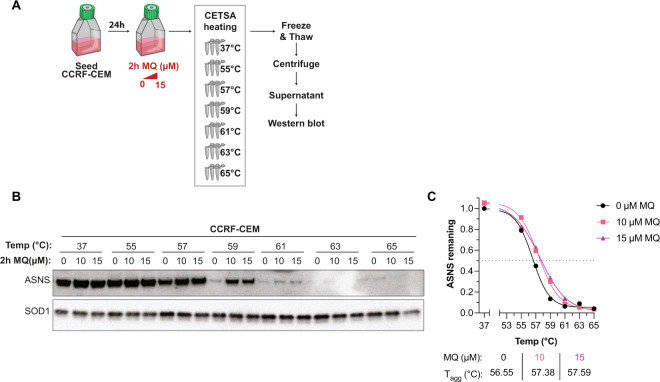
MQ thermostabilizes ASNS in ALL cells. **A** Overview of experimental set-up for MQ treatment of CCRF-CEM cells and WB-CETSA 2h after treatment. **B** WB-CETSA of CCRF-CEM cells treated for 2 h with the indicated concentrations of MQ and heated at the indicated temperature as shown in **A**. SOD1 was used as is a thermally stable loading control. *n* = 1. **C** Quantification of WB-CETSA in **B**. ASNS signal was normalized to loading control SOD1 in the same samples. All values were normalized to 37 °C without MQ. Aggregation temperature (*T*_agg_) was determined at 50% remaining ASNS according to the Boltzmann sigmoidal curve. *n* = 1.

Analysis of pharmacogenomic datasets available through the DepMap portal [37] with >300 cancer cell lines of various origins did not show any significant difference in PRIMA-1 (APR-246 analog) sensitivity between cells with high and low ASNS expression (Fig. S2A). Thus, the effect of APR-246/MQ on ASNS activity may not be biologically relevant in a pan-cancer setting. However, as resistance to ASNase in ALL is associated with increased ASNS expression [15, 19–22], targeting ASNS has therapeutic relevance in ALL [13]. Therefore, we first sought to validate MQ-induced thermal stabilization of ASNS in the ALL cell line CCRF-CEM. Western blot-based CETSA (WB-CETSA) was performed as previously described (Fig. 1A) but with 10 and 15 µM MQ and additional temperatures around 58 °C to achieve a full melting curve (Fig. 2A) where the ASNS shift was observed in the MS-CETSA (Fig. 1E). WB-CETSA did not show any change in ASNS protein level at 37 °C but both concentrations of MQ induced a temperature-dependent stabilization of ASNS at 57 and 59 °C (Fig. 2B and S2B). SOD1 was used as thermally stable loading control as described [38] and was unaffected by treatment and temperature. Ponceau staining indicated temperature-dependent protein degradation while protein loading within the same temperature was similar (Fig. S2B). Quantification of the WB-CETSA showed a thermal shift of ASNS from 56.6 to 57.5 °C upon incubation with MQ at the aggregation temperature (*T*_agg_) when half of the protein has aggregated and been removed from the soluble fraction (Fig. 2C and S2C). Thus, WB-CETSA in a tumor model relevant to ALL biology indicates that MQ, the active product of APR-246, directly or indirectly modulates ASNS.

### Markers for ASNase and APR-246 sensitivity in ALL cells

**Fig. 3:**
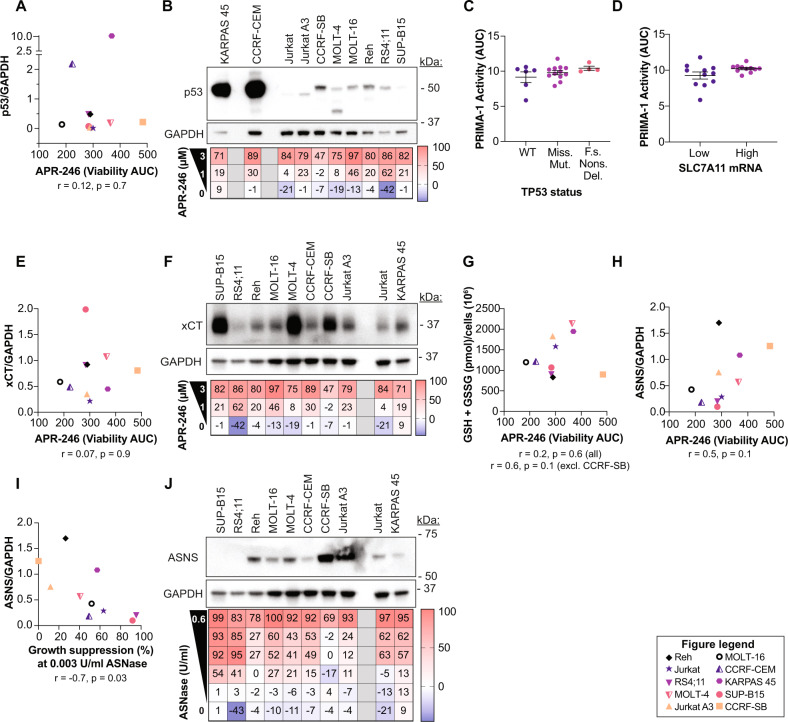
Markers for ASNase and APR-246 sensitivity in ALL cells. **A** p53 protein levels in untreated cells plotted against cell viability after 72 h treatment with APR-246. p53 levels were obtained by quantification of the western blot in **B**. Cell viability was assessed by resazurin (area under the curve [AUC]). See Table S5. Spearman correlation *r* = 0.12, *p* = 0.7. **B** Western blot of untreated ALL cell lines and a heat map indicating growth suppression as assessed by resazurin after 72 h of APR-246 treatment. **C** Analysis of data from the DepMap portal of the Broad Institute of 22 ALL cancer cell lines showing PRIMA-1 activity area under the curve (AUC) grouped into *TP53* status. WT = wild type, Miss. Mut. = missense mutation, F.s. = frameshift, Nons. = nonsense, Del. = deletion **D** Analysis of data from the DepMap portal of the Broad Institute of 22 ALL cancer cell lines showing PRIMA-1 activity area under the curve (AUC) grouped into xCT/SLC7A11 mRNA expression below or above median. Unpaired *t* test, *p* = 0.08. **E** xCT protein levels in untreated cells plotted against cell viability after 72 h treatment with APR-246. xCT levels were obtained by quantification of the western blot in **F**. Cell viability was assessed by resazurin (area under the curve [AUC]). See Table S5. Spearman correlation *r* = 0.07, *p* = 0.9. **F** Western blot of untreated ALL cell lines and a heat map indicating growth suppression as assessed by resazurin after 72 h of APR-246 treatment (same as in **B** but in different order). **G** Total glutathione (GSH + GSSG) measured by enzymatic recycling assay in untreated ALL cell lines after 72 h in culture plotted against cell viability after 72 h of APR-246 treatment. Cell viability was assessed resazurin (area under curve [AUC]). Cell lines are specified in the figure legend box. See Table S5. Spearman correlation *r* = 0.2, *p* = 0.6 or excluding CCRF-SB *r* = 0.6, *p* = 0.1. **H** ASNS protein levels in untreated cells plotted against cell viability after 72 h treatment with APR-246. ASNS levels were obtained by quantification of the western blot in **J**. Cell viability was assessed by resazurin (area under the curve [AUC]). See Table S5. Spearman correlation *r* = 0.5, *p* = 0.1. **I** ASNS protein levels in untreated cells plotted against growth suppression after 72 h treatment with 0.003 U/ml ASNase. ASNS levels were obtained by quantification of the western blot in **J**. Growth suppression (%) was assessed by resazurin. See Table S4. Spearman correlation *r* = −0.7, *p* = 0.03. **J** Western blot of untreated ALL cell lines and a heat map indicating growth suppression as assessed by resazurin after 72 h of asparaginase (ASNase) treatment. Detailed information and *n* regarding growth suppression are given in Table S4. The same GAPDH control blot is shown in panels **F** and **J** since both xCT (**F**) and ASNS (**J**) were examined on the same blot. The blot was divided into an upper part which was probed with ASNS antibody and a lower part which was probed with xCT antibody and then re-probed with GAPDH antibody. Thus, the same GAPDH blot serves as control for both xCT and ASNS.

Demir et al. demonstrated increased APR-246 efficacy in mutant *TP53* ALL cell lines and patient-derived ALL xenografts in mice [32], in line with many of our previous studies showing that cancer cells with mutant *TP53* are more sensitive to APR-246 than wild-type *TP53* cells [6, 7, 11, 39]. However, other studies have demonstrated p53-independent sensitivity to APR-246 [40, 41]. Furthermore, we have recently shown that neither *TP53* status, GSH content, nor drug accumulation alone can fully determine APR-246 sensitivity [11]. We did not detect any correlation between p53 protein expression levels and APR-246 sensitivity in the ten ALL cell lines included in this study (Figs. 3A, B and S3A). For example, KARPAS-45 and CCRF-CEM with hot spot *TP53* mutations (Table S2) and very high levels of mutant p53 expression were only moderately sensitive to APR-246 and their half-maximal inhibitory concentration (IC_50_) values (Table S3) did not differ from those of other ALL lines with lower levels of p53 expression. Interestingly, CCRF-SB that carries wild-type *TP53* was highly resistant to APR-246 (Table S3). Database analysis of 22 ALL cell lines in the DepMap portal did not show any difference in PRIMA-1 sensitivity depending on *TP53* status (Fig. 3C) as has also been observed in a Pan-Cancer setting and overall in tumors of hematological lineages [42].

MQ can trigger p53-independent cell death by induction of oxidative stress [6, 40, 43]. This effect can be inhibited by various antioxidative compounds and cellular mechanisms. For example, expression levels of the cystine/glutamate antiporter xCT (*SLC7A11*), which imports glutathione building blocks, shows one of the highest correlations to PRIMA-1 resistance in a DepMap analysis of >700 cancer cell lines [11] and has been suggested as a predictive biomarker for APR-246 sensitivity [42, 44]. Surprisingly, when stratifying for only ALL cell lines in the DepMap database, we found no difference in PRIMA-1 sensitivity between ALL cell lines with high and low xCT/SLC7A11 mRNA levels (Fig. 3D). Similarly, there was no correlation between APR-246 sensitivity and xCT protein levels among the ten ALL cell lines included in the study (Fig. 3E, F) nor with antioxidant TrxR1 protein levels (Fig. S3B). Antioxidant capacity reflected by total glutathione (GSH + GSSG) did show a weak correlation with APR-246 sensitivity (*r* = 0.6, *p* = 0.1) but only when the APR-246-resistant cell line CCRF-SB (IC_50_ > 9 μM) was excluded (Figs. 3G and S3C, D). The DepMap analysis also did not show any significant correlation between reduced glutathione (GSH) and PRIMA-1 activity in 22 cell lines of ALL origin (Fig. S3E). Asparagine and PRIMA-1 activity (area under the curve (AUC)) did not correlate (Fig. S3F), but interestingly, aspartate was inversely correlated to PRIMA-1 activity in 22 ALL cell lines in the DepMap analysis (Fig. S3G) (*r* = −0.5, *p* = 0.03). Thus, ALL cells with high aspartate are more sensitive to PRIMA-1, while this correlation was not apparent in a pan-cancer setting (>700 cell lines) (Fig. S3H, I). Western blot quantification of ASNS indicated a weak correlation (*r* = 0.5, *p* = 0.1) between high ASNS expression and APR-246 resistance (Fig. 3H). This would be expected if MQ binding inhibits the enzyme, since more MQ will be needed to inhibit ASNS function if more enzyme is present.

In agreement with other studies [19–22], we observed a clear correlation (*r* = −0.7, *p* = 0.03) between low ASNS expression and high ASNase growth suppression (Fig. 3I, J). RS4;11 and SUP-B15 were the most sensitive lines to ASNase with no visible ASNS expression according to Western blot analysis, while CCRF-SB, Jurkat A3 cells, and Reh had low ASNase sensitivity with a high expression of ASNS. All other cell lines that had visible ASNS bands showed intermediate ASNase sensitivity. We conclude that ALL has a distinct metabolic landscape and that APR-246 sensitivity in ALL, unlike in solid tumors, is not correlated to mutant p53 or xCT expression levels.

### APR-246 synergizes with ASNase in ALL cells

**Fig. 4:**
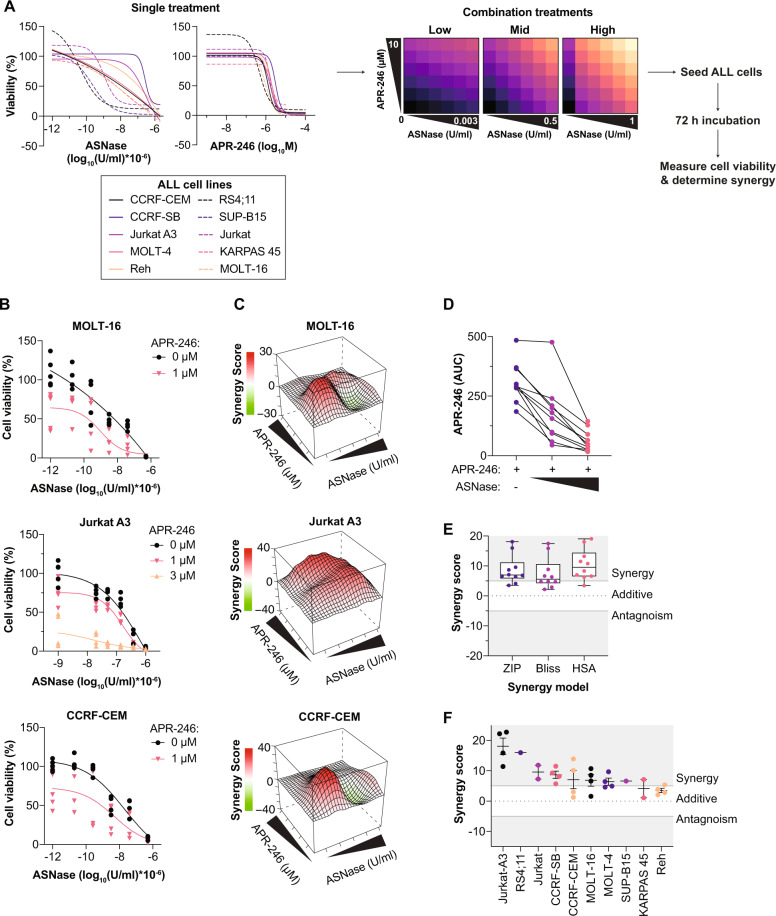
APR-246 synergizes with ASNase in ALL cells. **A** Single treatment (*n* = 2–7) and experimental overview of combination treatments. Cell viability as assessed by resazurin after 72 h single treatment with either asparaginase (ASNase) or APR-246. Three different ASNase treatment groups (low, mid, high) for the combination treatments were used; APR-246 concentrations were the same for all cell lines. **B** Cell viability as assessed by resazurin after 72 h of increasing concentrations of ASNase in combination with APR-246. Each dot indicates an individual experiment. **C** Synergy landscape determined by ZIP model over APR-246 and ASNase concentrations of one representative experiment. Concentrations of APR-246 and ASNase are as in Fig. S4A. **D** Cell viability (area under the curve [AUC]) as assessed by resazurin after 72 h of APR-246 alone or with two different concentrations of ASNase (0.04–0.05 and 0.4–0.5 U/ml for all cell lines except for RS4;11 and SupB15 that were treated with 0.009 and 0.003 U/ml). Each dot indicates one ALL cell line. See Table S5 for more details and *n* for each cell line. **E** Box-and-whisker plot of synergy scores of most synergistic area determined by three different synergy models. Central band indicates median, boxes indicated 25th and 75th percentile, and whiskers show min. and max. values. Each dot indicates one ALL cell line. See Table S6 for more details, including *n* for each cell line. **F** Synergy score of most synergistic area determined by the ZIP model in 10 different ALL cell lines after ASNase treatment +/− APR-246 for 72 h. Each dot indicates one experiment.

Given that ASNase resistance is associated with ASNS expression, and that our data suggest that APR-246/MQ may target ASNS, we hypothesized that APR-246 may sensitize ALL cells to ASNase treatment. After determining the effect of APR-246 or ASNase monotherapy, we measured cell viability in a panel of ten ALL cell lines following a 72-h treatment with a concentration response matrix consisting of APR-246 and three different ASNase concentrations ranges (low, mid, and high) (Fig. 4A). In agreement with another study [42] showing that hematological lineage tumor cells are more sensitive to APR-246 treatment than solid cancer cell lines, we observed that the APR-246 IC_50_ values (Table S3) in most of the tested ALL cell lines were more than tenfold lower compared to the IC_50_ values of multiple previously tested solid cancer cell lines [11]. Furthermore, incubation with APR-246 reduced cell viability after ASNase treatment in several of the tested ALL cell lines, for example, in MOLT-16 and Jurkat A3 with low p53 levels and CCRF-CEM cells with high p53 levels (Figs. 4B and S4A). We observed an increased growth suppression at several tested concentrations (Fig. S4B). Synergistic growth suppression was found at several concentration combinations as determined using the web application SynergyFinder over the concentration response matrices (Fig. 4C).

In general, APR-246 sensitivity was increased (as demonstrated by decreased AUC) in the combination with ASNase (Fig. 4D). According to the three tested synergy models, the combination of APR-246 and ASNase resulted in synergistic growth suppression (scores > 0; Fig. 4E). Eight out of ten tested ALL cell lines exhibited synergy, although to varying extent (Fig. 4F). We did not observe any correlation with p53, xCT, or ASNS expression and synergy scores (Fig. S4C–E). For example, Jurkat A3, Reh, and CCRF-SB, which are among the most ASNase-resistant cell lines with the highest ASNS expression (Fig. 3J), exhibited variable degrees of synergy scores (Fig. 4F), similar to the most ASNase-sensitive cell lines with no detectable ASNS expression, RS4;11 and SupB15.

In summary, combination treatment with APR-246 and ASNase results in synergistic growth suppression in several ALL cell lines independently of p53, xCT, and ASNS.

## Discussion

The mutant p53-targeting compound APR-246 is currently being tested in phase III clinical trials in patients with *TP53* mutant MDS and several phase I and II studies in other hematological malignancies with mutant *TP53* or solid cancers independent of *TP53* status. The prodrug APR-246 is converted to the Michael acceptor MQ that reactivates mutant p53 and also induces oxidative stress by targeting redox regulators such as TrxR1, thioredoxin, and glutathione, leading to tumor cell death by apoptosis [45]. Given the thiol-binding properties of MQ, we reasoned that additional MQ targets may play variable roles in APR-246-induced tumor cell death, depending on the cellular context.

Our CETSA screen demonstrated that ASNS is thermally stabilized in MQ-treated cells (Figs. 1 and 2). This could result from direct binding of MQ or indirect effects including, for instance, binding to another protein that is stabilized by MQ or from stabilizing posttranslational modification induced by MQ. Our data suggest that stabilization of ASNS is associated with inhibition of the enzyme, as indicated by the observed correlation between APR-246 efficacy and ASNS protein levels (Fig. 3H). Furthermore, MQ is a very reactive molecule with a preference for “soft” nucleophiles like the thiol of a cysteine [11], such as the cysteine in the N-terminal active site that was reported to be essential for the glutamine-dependent activity of ASNS [46]. Taken together, these data suggest that the thermal stabilization may be derived from MQ binding to this active site cysteine in ASNS, and if so, it is conceivable that MQ inhibits enzyme activity as demonstrated for TrxR1 [9] and other redox enzymes [10].

**Fig. 5:**
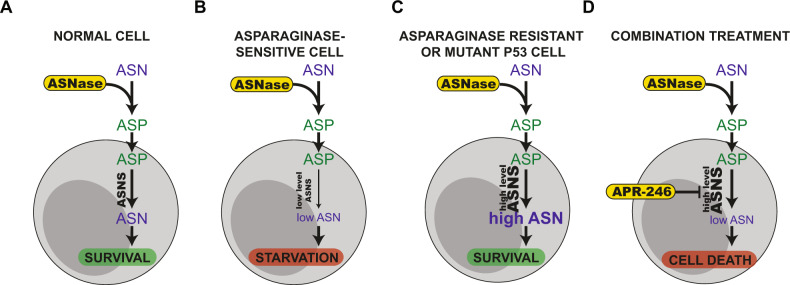
Proposed mechanisms of synergy between APR-246 and ASNase. **A** Asparaginase (ASNase) depletes extracellular asparagine (ASN) by converting it to aspartic acid (ASP). Normal cells can synthesize ASN by asparagine synthetase (ASNS). **B** Acute lymphoblastic leukemia (ALL) cells have defective ASNS expression and are therefore sensitive to ASNase treatment. **C** ASNase resistance is associated with ASNS expression and the resistant cells can therefore synthesize ASN from ASP. **D** Combination treatment with mutant p53 reactivating compound APR-246 directly or indirectly inhibits ASNS, resulting in enhanced sensitivity in ASNase-resistant ALL cells.

During the past decades, ASNase has been successfully used as standard-of-care treatment for ALL [12, 47]. For instance, in childhood ALL, recombinant *Escherichia coli* ASNase treatment alone can induce complete remission in up to 40–60% of the patients. This effect is largely attributed to ASNase-mediated depletion of circulating asparagine in the blood, by converting it to aspartate. However, it should be noted that ASNase also has glutaminase activity that contributes to antitumor effect [12]. Normal cells can import aspartate and synthesize asparagine intracellularly through ASNS (Fig. S1F). Asparagine is important for tumor growth as a substrate for protein synthesis and due to its role in cellular amino acid homeostasis [48] (Fig. 5), but it may also have other regulatory functions [49]. ASNS is part of the amino acid response (AAR) pathway with AAR elements and nutrient-sensing response elements in its promoter. Consequently, upon asparagine depletion (but also depletion of other amino acids), ASNS will be upregulated via activating transcription factor 4 (ATF4) [50, 51]. ALL cells are asparagine auxotrophs, relying on circulating asparagine, and are therefore highly sensitive to ASNase treatment (Fig. 5B). Although ASNS mRNA is upregulated upon asparagine deprivation [21], the protein is not expressed [49], possibly as a result of promoter methylation [52] or epigenetic changes [53] in ALL cells. ASNS upregulation is one mechanism of ASNase resistance, enabling cells to synthesize aspartate and thereby survive and grow even when circulating asparagine is depleted [12, 19] (Fig. 5C).

Although *TP53* mutations are infrequent in ALL overall, *TP53* is mutated at a higher frequency in relapsed ALL and this is associated with poor therapy response [4, 24, 25]. Wild-type p53 can downregulate ASNS transcription [54] and mutant p53 can upregulate ASNS expression by binding to its promoter according to another study [55]. This suggests that mutant p53 could play a role in ASNase resistance (Fig. 5C). In support of this notion, our TCGA-PanCancer analysis demonstrated that patients with wild-type *TP53* show lower ASNS expression compared to patients with missense or truncating *TP53* mutations (Fig. S4F).

Apart from ALL, ASNase has been used for treatment of other hematological malignancies including AML [17], for which APR-246 has received FDA fast track designation. This raises interesting future perspectives for combination treatment. A screen of >900 cancer cell lines identified aberrant ASNS promoter hypermethylation resulting in lack of ASNS protein expression in gastric and hepatic cancer cell lines. Mouse xenograft models with these cell lines showed high sensitivity to ASNase treatment [56]. As *TP53* mutation is common in gastric cancer [57] and a driver mutation and frequent event (> 30%) in hepatic cancers [58], the combined targeting of mutant p53 with APR-246 and depletion of asparagine with ASNase may also be fruitful approach in some solid cancers with low ASNS expression.

Another interesting tumor type for the combination treatment with APR-246 and ASNase is *KRAS*-driven non-small-cell lung cancer (NSCLC). Around 20% of these tumors carry mutation in Kelch-like ECH-associated protein 1 (KEAP1) or NRF2 [59]. Tumors with mutation in master antioxidant response regulator NRF2 or its negative regulator KEAP1 have high antioxidant capacity, for example, via induction of antiporter xCT (SLC7A11) expression, resulting in efflux of glutamate and influx of cystine, which may be used for antioxidant glutathione synthesis. LeBoeuf et al. demonstrated that xCT-driven glutamate efflux makes KEAP1-mutated tumors ASNase sensitive as they rely on the uptake of non-essential amino acids, including asparagine [59]. Additionally, high xCT activity renders tumors glutamine dependent as glutamine is utilized for generating glutamate that is exported with the imported cystine [60–62]. This may increase their sensitivity to the glutaminase activities of ASNase [16]. Both xCT [44] and KEAP1 [42] are correlated to APR-246 resistance, and so it is conceivable that ASNase may sensitize APR-246-resistant tumors. Furthermore, wild-type KEAP1 tumor cells may be sensitized to ASNase treatment by induction of oxidative stress [59]. Thus, as APR-246 (via MQ) depletes glutathione (Fig. 1B), APR-246 may also sensitize NSCLC without KEAP1 mutations to ASNase treatment.

Our data indicate that the mutant p53 reactivator APR-246, currently in phase III clinical trials in MDS, targets ASNS and thereby possibly inhibits its activity. Mutant p53 reactivation by APR-246 has previously been demonstrated in patient-derived ALL xenografts in mice [32]. The mutant p53-reactivating and ASNS-targeting activities of APR-246 might exert a dual effect on ASNS-expressing cells (Fig. 5D), resulting in synergistic growth suppression upon combination treatment with ASNase. Since *TP53* mutation is associated with poor treatment response and ASNS expression is associated with ASNase resistance, targeting both mutant p53 and ASNS with APR-246 in combination with ASNase, which depletes circulating asparagine, appears as an attractive strategy that warrants further investigation in ALL, especially in light of the benign safety profile of APR-246 [63].

## Materials and methods

### Cell lines and culture conditions

OVCAR-3, RS4;11, SupB15, and KARPAS-45 were cultured in RPMI-1640 media containing Hepes (HyClone). Insulin–Transferrin–Selenium (51300044, Thermo Fisher Scientific) was freshly added into the media for OVCAR-3. Molt-16 was cultured in Iscove’s Modified Dulbecco’s Medium (Hyclone) media. Molt-4, Reh, Jurkat, Jurkat A3, CCRF-CEM, and CCRF-SB were cultured in RPMI-1640 media All media were supplemented with L-glutamine and 10% fetal bovine serum. Cell count and viability were determined by Countess^TM^ II Automated Cell Counter (Thermo Fisher Scientific).

### Drugs and reagents

APR-246 and MQ were provided by Aprea Therapeutics. APR-246 was prepared in 100 mM stock in dimethyl sulfoxide (DMSO) stored at −20 °C. MQ was prepared in 50 mM H_2_O stocks and stored at −80 °C. ASNase from *E. coli* (ASNase) was purchased from Sigma (A3809-100UN) and prepared in milliQ H_2_O to a stock concentration of 500 UN/ml stored at −20 °C. For treatment using Tecan 300d, ASNase was diluted in 0.3% Tween-20 before adding to plates. Unless stated otherwise, all reagents mentioned hereafter were from Merck (Germany).

### MS-based CETSA

OVCAR-3 (1.8 × 10^6^ cells) were seeded per 10 cm dish. The next day media was changed into 12 ml of culturing media containing 0.0025, 0.01, 0.05, 0.2, 0.8, 3.1, 12.5, 50, or 200 µM MQ prepared in duplicates. Control samples were only media. Cells were incubated for 2 h in 37 °C at 5% CO_2_. After incubation, the media was poured out and cells were washed once with phosphate-buffered saline (PBS) and incubated with 2 ml trypsin (HyClone) at 37 °C. When cells detached, 5 ml Hank’s Balanced Salt Solution (HBSS) (14175-053, Thermo Fisher Scientific) was added and cells were centrifuged 300 g for 5 min. Supernatant was discarded and cells were resuspended in HBSS. Each treatment condition (in duplicates) was aliquoted into four different PCR stripes (for four different temperatures) in 100 µl volumes. Leftover aliquot was used to determine cell number and viability by trypan blue staining. The PCR stripes were transferred to PCR machine Veriti thermal cycler (Applied Biosystems) and heated for 3 min at 37, 42, 52, or 58 °C after which the samples were cooled down on ice and 1 µl of Halt^TM^ Protease Inhibitor Cocktail 100×, EDTA free (78429 Thermo Fisher Scientific) was added to each sample. Samples were vortexed and snap frozen by liquid nitrogen and then thawed in 20 °C in the PCR machine, this was repeated twice. Lysates were centrifuged at 20,000 × *g* for 20 min at 4 °C and then 80 µl of the supernatant was moved to new tubes on a PCR plate and were frozen at −80 °C.

Samples were thawed and protein concentration was determined by the Pierce^TM^ BCA Protein Assay Kit (Thermo Scientific). Due to high cell death (Fig. 1C) and low protein concentration, the 50 and 200 µM MQ samples were not prepared for MS-CETSA. Equal amounts of total protein from each condition were prepared for MS analysis as previously described [30]. In brief, samples were dried and resuspended in 50 mM triethylammonium bicarbonate buffer (T7408, Sigma) in H_2_O (liquid chromatography (LC)-MS grade, 115333, Merck), reduced with 5 mM tris(2-carboxyethyl)phosphine (77720, Bond-breaker^TM^, Thermo Scientific) at 65 °C for 30 min, followed by alkylation with 15 mM 2-chloroacetamide (C0267, Sigma) at 37 °C in the dark. Next, samples were digested with 1:50 Lys-C (Wako Chemicals Ltd) at 37 °C for 2 h, followed by 1:50 SOLu-Trypsin (EMS0004, Sigma) at 37 °C overnight after which efficiency was checked. Isobaric Tandem Mass Tags (TMT)−10plex (90110, Thermo Scientific) at 37 °C for 3 h was used for labeling peptides and labeling efficiency (>95% TMT-labeled peptide spectrum matches (PSMs)) was checked. Samples from one experiment in duplicates from the treatment conditions at the same temperature were labeled as a set. Next, the labeled samples from the same TMT set were pooled. Labeling reaction was stopped by adding 10% trifluoroacetic acid (Sigma) to reach pH <3 and samples were dried and desalted using Oasis HLB 1 cc (10 mg) extraction cartridges (186000383, Waters) according to the manufacturer’s protocol. Offline pre-fractionation of the samples was performed by high pH reverse-phase LC using the ÄKTA Micro system (GE Healthcare) using a Xbridge Peptide BEH C18, 300 Å, 3.5 µm, 2.1 mm × 250 mm column (#186003610, Waters). The fractions were concentrated into 12 fractions, dried, and resuspended in 0.1% formic acid (FA; LC-MS grade, 533002, Merck) in H_2_O (LC-MS grade, 115333 Merck) for online chromatography, performed using Dionex UltiMate 3000 UPLC system coupled to a Q Exactive HF mass spectrometer (Thermo Scientific). Using a 50 cm × 75 μm (ID) EASY-Spray analytical column (Thermo Scientific) in 70 min gradient of programmed mixture of solvent A (0.1% FA in H_2_O) and solvent B (99.9% acetonitrile, 0.1% FA), each fraction was separated. MS data were acquired using a top 12 data-dependent acquisition method. Full-scan MS spectra were acquired in the range of 300–600 *m*/*z* at a resolution of 70,000 and AGC target of 3e6: top 12 dd-MS2 70,000, and 3e6 with isolation window at 1.2 *m*/*z*.

### Protein identification, quantification, hit selection, and data visualization

Raw MS-CETSA data were analyzed using Proteome Discoverer 2.1 (Thermo Fisher Scientific) for peptide and protein identification and quantification using Mascot 2.6.0 (Matrix Science) and Sequest HT (Thermo Fisher Scientific). Peptide sequences were then mapped against human proteome database from Uniprot with the following search parameters: trypsin, missed cleavage sites <3, precursor mass tolerance 20 ppm, fragment mass tolerance 0.05 Da. The search included dynamic modifications (acetylation [protein N-terminus], oxidation [M], deamidation [NQ]) as well as static modifications (carbamidomethylation [C], TMT10-plex [K and peptide N-terminus]). A threshold for false discovery rate of 1% was set for PSM and peptide levels. Unique and razor peptides were used for protein assignment and abundance quantification and only master proteins in the protein group were used for further downstream data analysis and visualization by our in-house developed mineCETSA package [34] in RStudio. Abundance data were extracted, cleaned up, and a systematic scaling step was performed, followed by curve fitting and plotting. Measured relative fold changes as compared to vehicle control at different temperatures reflect protein thermal stability changes upon compound treatment. Only target proteins with at least three PSM and a minimum fold change of 30% as compared to vehicle were included for hit selection. Additionally, only hits showing thermal stabilization in 46, 52, or 58 °C but not in 37 °C were considered for hit selection and sorted by AUC.

### Western blot-based CETSA

CCRF-CEM cells were counted, centrifuged at 300 × *g* for 5 min, and resuspended into new media at a dilution of 0.8 × 10^6^ cells/ml. Cells were aliquoted into 15 ml of cell suspension per standing 25-cm^2^ flask and incubated at 37 °C and 5% CO_2_. The next day MQ was added into the media to a final concentration of 10 or 15 µM of MQ and incubated for 2 h at 37 °C and 5% CO_2_; control cells were left untreated. After 2 h, cells were collected and centrifuged for 3 min at 300 × *g*, washed with HBSS, and counted by trypan blue and the cell counter. Cells were centrifuged for 3 min at 300 × *g*, resuspended to 60 × 10^6^ cells/ml in HBSS, and aliquoted into 50 µl/PCR tubes into 7 different PCR stripes (7 different temperatures). The PCR stripes were transferred to PCR machine Veriti thermal cycler (Applied Biosystems) and heated for 3 min at the indicated temperatures (37–65 °C) after which the samples were cooled down on ice and 0.5 µl of 100× Protease Inhibitor Cocktail in DMSO (P8340, Merck/Sigma-Aldrich) was added to each sample. Samples were vortexed and snap frozen by liquid nitrogen and then thawed in 20 °C in the PCR machine; this was repeated twice. Lysates were centrifuged at 20,000 × *g* for 20 min at 4 °C and then 40 µl of the supernatant was moved to new PCR tubes and were frozen at −80 °C.

### Western blotting

Western blot was performed as previously described [11]. Equal amount of protein was boiled in LDS sample buffer containing reducing agent while for WB-CETSA 10 µl of protein lysate from each sample was used. Primary antibodies used were horseradish peroxidase (HRP)-conjugated antibody against p53 (DO-1) diluted 1:5000 (sc-126 HRP, Santa Cruz Biotechnology, USA) and GAPDH diluted 1:30,000 (sc-47724 HRP, Santa Cruz Biotechnology, USA). Primary antibodies without any conjugation were against ASNS diluted 1:1000 (sc-365809, Santa Cruz Biotechnology, USA), SOD1 diluted 1:20,000 (HPA001401, Sigma), xCT/SLC7A11 (D2M7A) diluted 1:1000 (12691S, Cell Signaling, USA), and TrxR1 diluted 1:1000 (Santa Cruz Biotechnology, USA).

### Cell viability measurement by resazurin

Drug combination effects were determined by resazurin cell viability measurements were performed as described previously [64, 65]. ALL cells were diluted to 2 × 10^4^ live cells/ml in fresh media. APR-246 (100 mM in DMSO) and ASNase (250 U/ml in 0.3% Tween-20) were dispensed into clear bottomed 384-well plates (Corning) using the D300e digital dispenser (Tecan), and solvents were normalized across the plates. Subsequently, cells were seeded in 50 µl/well using a MultiDrop (Thermo Fisher) to give a final seeding concentration of 1000 cells/well. Plates were incubated at 37 °C and 5% CO_2_ for 72 h in a humidity chamber, until Resazurin (R17017, Sigma Aldrich) dissolved in PBS was added to a final concentration of 0.01 mg/ml. After 4–6 h of Resazurin incubation, fluorescence at 530/590 nm (ex/em) was measured by Hidex Sense Microplate Reader. Wells containing only media and solvent were used to subtract background, and data were normalized to vehicle-treated wells.

### Total glutathione (GSH + GSSG) measurements by enzymatic re-cycling assay

ALL cells were seeded into standing 25-cm^2^ cell culturing flasks (Sarstedt) in 10 ml media at a cell density of 0.2 × 10^6^ cells/ml except RS4;11 and SupB15, which were seeded at double density due to lower proliferation rate. After 24 or 72 h of incubation at 37 °C and 5% CO_2_, cells were harvested by 5 min 500 × *g* centrifugation, washed in PBS, counted, centrifuged, and resuspended in 100 µl of 10 mM HCl. Samples were centrifuged at 20,800 × *g* for 20 min at 4 °C, 60 µl of the supernatant was transferred to new tubes, and frozen at −20 °C. Samples were thawed to measure total GSH + GSSG as described [11].

### Glutathione Reductase (GR) activity assay

Recombinant GR activity assays were performed as described [11].

### Confluence assessment by Incucyte

The day before treatment, 3000 OVCAR-3 cells were seeded per well in a 96-well plate with 100 µl media. The next day, indicated treatments were added from a diluted stock into the media and incubated in the IncuCyte® S3 real-time video imaging system (Essen BioScience, USA) stored in a cell incubater at 37 °C and 5% CO_2_. Caspase-3 dye was incubated at the same time as drug treatments. Each well was imaged every third hour up to 72 h in duplicate wells and 4 images per well. Confluence was based on bright-field images and caspase 3 activity was assessed by measuring green fluorescence and normalized to confluence in each image. Confluence analysis was performed by the Incucyte software and normalized to starting time point for each condition.

### Data analysis and statistics

Results presented in figures are mean and standard error of the mean unless otherwise specified. Adobe Illustrator was used to put together figure panels and draw illustrative figures. GraphPad Prism 9 was used to prepare heat maps, histograms, scatter plots, and perform statistical analysis. Normal distribution of dataset was tested by Shapiro–Wilks test, and depending on the outcome, parametric or nonparametric statistical tests were performed. Data for correlation analysis of PRIMA-1, gene expression, and metabolites were downloaded from Cancer Dependency Map (https://www.depmap.org) [56, 66, 67] and analyzed in GraphPad Prism 9. Synergy was determined as previously described [11] using SynergyFinder web application 2.0 by FIMM (https://synergyfinder.fimm.fi/) using ZIP, Bliss, or HSA synergy [68].

## Supplementary information

Supplementary Tables

Figure S1

Figure S2

Figure S3

Figure S4

Supplementary Figure Legends

## References

[1] Kandoth C, McLellan MD, Vandin F, Ye K, Niu B, Lu C (2013). Mutational landscape and significance across 12 major cancer types. Nature..

[2] Boettcher S, Miller PG, Sharma R, McConkey M, Leventhal M, Krivtsov AV (2019). A dominant-negative effect drives selection of TP53 missense mutations in myeloid malignancies. Science..

[3] Bally C, Ades L, Renneville A, Sebert M, Eclache V, Preudhomme C (2014). Prognostic value of TP53 gene mutations in myelodysplastic syndromes and acute myeloid leukemia treated with azacitidine. Leuk Res.

[4] Hof J, Krentz S, van Schewick C, Korner G, Shalapour S, Rhein P (2011). Mutations and deletions of the TP53 gene predict nonresponse to treatment and poor outcome in first relapse of childhood acute lymphoblastic leukemia. J Clin Oncol.

[5] Mantovani F, Collavin L, Del Sal G (2019). Mutant p53 as a guardian of the cancer cell. Cell Death Differ.

[6] Lambert JM, Gorzov P, Veprintsev DB, Soderqvist M, Segerback D, Bergman J (2009). PRIMA-1 reactivates mutant p53 by covalent binding to the core domain. Cancer Cell.

[7] Bykov VJ, Issaeva N, Shilov A, Hultcrantz M, Pugacheva E, Chumakov P (2002). Restoration of the tumor suppressor function to mutant p53 by a low-molecular-weight compound. Nat Med.

[8] Mohell N, Alfredsson J, Fransson A, Uustalu M, Bystrom S, Gullbo J (2015). APR-246 overcomes resistance to cisplatin and doxorubicin in ovarian cancer cells. Cell Death Dis.

[9] Peng X, Zhang MQ, Conserva F, Hosny G, Selivanova G, Bykov VJ (2013). APR-246/PRIMA-1MET inhibits thioredoxin reductase 1 and converts the enzyme to a dedicated NADPH oxidase. Cell Death Dis.

[10] Haffo L, Lu J, Bykov VJN, Martin SS, Ren X, Coppo L (2018). Inhibition of the glutaredoxin and thioredoxin systems and ribonucleotide reductase by mutant p53-targeting compound APR-246. Sci Rep.

[11] Ceder S, Eriksson SE, Cheteh EH, Dawar S, Corrales Benitez M, Bykov VJN, et al. A thiol-bound drug reservoir enhances APR-246-induced mutant p53 tumor cell death. EMBO Mol Med. 2020;13:e10852. 10.15252/emmm.201910852 PMC7863383 33314700

[12] Lanvers-Kaminsky C (2017). Asparaginase pharmacology: challenges still to be faced. Cancer Chemother Pharmacol.

[13] Richards NG, Kilberg MS (2006). Asparagine synthetase chemotherapy. Annu Rev Biochem.

[14] Lazarus H, McCoy TA, Farber S, Barell EF, Foley GE (1969). Nutritional requirements of human leukemic cells. Asparagine requirements and the effect of L-asparaginase. Exp Cell Res.

[15] Prager MD, Bachynsky N (1968). Asparagine synthetase in normal and malignant tissues: correlation with tumor sensitivity to asparaginase. Arch Biochem Biophys.

[16] Parmentier JH, Maggi M, Tarasco E, Scotti C, Avramis VI, Mittelman SD (2015). Glutaminase activity determines cytotoxicity of L-asparaginases on most leukemia cell lines. Leuk Res.

[17] Emadi A, Zokaee H, Sausville EA (2014). Asparaginase in the treatment of non-ALL hematologic malignancies. Cancer Chemother Pharmacol.

[18] DeBerardinis RJ, Mancuso A, Daikhin E, Nissim I, Yudkoff M, Wehrli S (2007). Beyond aerobic glycolysis: transformed cells can engage in glutamine metabolism that exceeds the requirement for protein and nucleotide synthesis. Proc Natl Acad Sci USA.

[19] Gutierrez JA, Pan YX, Koroniak L, Hiratake J, Kilberg MS, Richards NG (2006). An inhibitor of human asparagine synthetase suppresses proliferation of an L-asparaginase-resistant leukemia cell line. Chem Biol.

[20] Haskell CM, Canellos GP (1969). L-asparaginase resistance in human leukemia−asparagine synthetase. Biochem Pharmacol.

[21] Aslanian AM, Fletcher BS, Kilberg MS (2001). Asparagine synthetase expression alone is sufficient to induce l-asparaginase resistance in MOLT-4 human leukaemia cells. Biochem J.

[22] Kiriyama Y, Kubota M, Takimoto T, Kitoh T, Tanizawa A, Akiyama Y (1989). Biochemical characterization of U937 cells resistant to L-asparaginase: the role of asparagine synthetase. Leukemia..

[23] Fine BM, Kaspers GJ, Ho M, Loonen AH, Boxer LM (2005). A genome-wide view of the in vitro response to l-asparaginase in acute lymphoblastic leukemia. Cancer Res.

[24] Comeaux EQ, Mullighan CG. TP53 mutations in hypodiploid acute lymphoblastic leukemia. Cold Spring Harbor Perspect Med. 2017;3:a026286. 10.1101/cshperspect.a026286 PMC5334249 28003275

[25] van Leeuwen FN (2020). Therapeutic targeting of mutated p53 in acute lymphoblastic leukemia. Haematologica..

[26] Almqvist H, Axelsson H, Jafari R, Dan C, Mateus A, Haraldsson M (2016). CETSA screening identifies known and novel thymidylate synthase inhibitors and slow intracellular activation of 5-fluorouracil. Nat Commun.

[27] Martinez Molina D, Jafari R, Ignatushchenko M, Seki T, Larsson EA, Dan C (2013). Monitoring drug target engagement in cells and tissues using the cellular thermal shift assay. Science..

[28] Jafari R, Almqvist H, Axelsson H, Ignatushchenko M, Lundback T, Nordlund P (2014). The cellular thermal shift assay for evaluating drug target interactions in cells. Nat Protoc.

[29] Sun W, Dai L, Yu H, Puspita B, Zhao T, Li F (2019). Monitoring structural modulation of redox-sensitive proteins in cells with MS-CETSA. Redox Biol.

[30] Dai L, Zhao T, Bisteau X, Sun W, Prabhu N, Lim YT (2018). Modulation of protein-interaction states through the cell cycle. Cell..

[31] Prabhu N, Dai L, Nordlund P (2020). CETSA in integrated proteomics studies of cellular processes. Curr Opin Chem Biol.

[32] Demir S, Boldrin E, Sun Q, Hampp S, Tausch E, Eckert C (2020). Therapeutic targeting of mutant p53 in pediatric acute lymphoblastic leukemia. Haematologica..

[33] Drake WR, Hou CW, Zachara NE, Grimes CL (2018). New use for CETSA: monitoring innate immune receptor stability via post-translational modification by OGT. J Bioenerg Biomembr.

[34] Lim YT, Prabhu N, Dai L, Go KD, Chen D, Sreekumar L (2018). An efficient proteome-wide strategy for discovery and characterization of cellular nucleotide-protein interactions. PLoS ONE.

[35] Rojo de la Vega M, Chapman E, Zhang DD. NRF2 and the hallmarks of cancer. Cancer Cell. 2018;34:21–43. 10.1016/j.ccell.2018.03.022 PMC6039250 29731393

[36] Eriksson SE, Ceder S, Bykov VJN, Wiman KG. p53 as a hub in cellular redox regulation and therapeutic target in cancer. J Mol Cell Biol. 2019;11:330–41. 10.1093/jmcb/mjz005 PMC6734141 30892598

[37] Ghandi M, Huang FW, Jane-Valbuena J, Kryukov GV, Lo CC, McDonald ER (2019). Next-generation characterization of the Cancer Cell Line Encyclopedia. Nature..

[38] Miettinen TP, Bjorklund M (2014). NQO2 is a reactive oxygen species generating off-target for acetaminophen. Mol Pharm.

[39] Bykov VJ, Issaeva N, Selivanova G, Wiman KG (2002). Mutant p53-dependent growth suppression distinguishes PRIMA-1 from known anticancer drugs: a statistical analysis of information in the National Cancer Institute database. Carcinogenesis.

[40] Tessoulin B, Descamps G, Moreau P, Maiga S, Lode L, Godon C (2014). PRIMA-1Met induces myeloma cell death independent of p53 by impairing the GSH/ROS balance. Blood..

[41] Grellety T, Laroche-Clary A, Chaire V, Lagarde P, Chibon F, Neuville A (2015). PRIMA-1(MET) induces death in soft-tissue sarcomas cell independent of p53. BMC Cancer.

[42] Fujihara KM, Corrales-Benitez M, Cabalag CS, Zhang BZ, Ko HS, Liu DS, et al. SLC7A11 is a superior determinant of APR-246 (Eprenetapopt) responsethan TP53 mutation status. Molecular Cancer Therapeutics, in press (accepted June 10, 2021) Available from: https://www.biorxiv.org/content/10.1101/2020.11.29.398875v1 10.1158/1535-7163.MCT-21-0067 34315763

[43] Ogiwara H, Takahashi K, Sasaki M, Kuroda T, Yoshida H, Watanabe R (2019). Targeting the vulnerability of glutathione metabolism in ARID1A-deficient cancers. Cancer Cell.

[44] Liu DS, Duong CP, Haupt S, Montgomery KG, House CM, Azar WJ (2017). Inhibiting the system xC-/glutathione axis selectively targets cancers with mutant-p53 accumulation. Nat Commun.

[45] Bykov VJN, Eriksson SE, Bianchi J, Wiman KG (2018). Targeting mutant p53 for efficient cancer therapy. Nat Rev Cancer.

[46] Van Heeke G, Schuster SM (1989). The N-terminal cysteine of human asparagine synthetase is essential for glutamine-dependent activity. J Biol Chem.

[47] Hoelzer D, Bassan R, Dombret H, Fielding A, Ribera JM, Buske C (2016). Acute lymphoblastic leukaemia in adult patients: ESMO Clinical Practice Guidelines for diagnosis, treatment and follow-up. Ann Oncol.

[48] Krall AS, Xu S, Graeber TG, Braas D, Christofk HR (2016). Asparagine promotes cancer cell proliferation through use as an amino acid exchange factor. Nat Commun.

[49] Chiu M, Taurino G, Bianchi MG, Kilberg MS, Bussolati O (2019). Asparagine synthetase in cancer: beyond acute lymphoblastic leukemia. Front Oncol.

[50] Ye J, Kumanova M, Hart LS, Sloane K, Zhang H, De Panis DN (2010). The GCN2-ATF4 pathway is critical for tumour cell survival and proliferation in response to nutrient deprivation. EMBO J.

[51] Chen H, Pan YX, Dudenhausen EE, Kilberg MS (2004). Amino acid deprivation induces the transcription rate of the human asparagine synthetase gene through a timed program of expression and promoter binding of nutrient-responsive basic region/leucine zipper transcription factors as well as localized histone acetylation. J Biol Chem.

[52] Ren Y, Roy S, Ding Y, Iqbal J, Broome JD (2004). Methylation of the asparagine synthetase promoter in human leukemic cell lines is associated with a specific methyl binding protein. Oncogene..

[53] Ding Y, Li Z, Broome JD (2005). Epigenetic changes in the repression and induction of asparagine synthetase in human leukemic cell lines. Leukemia.

[54] Deng L, Yao P, Li L, Ji F, Zhao S, Xu C (2020). p53-mediated control of aspartate-asparagine homeostasis dictates LKB1 activity and modulates cell survival. Nat Commun.

[55] Scian MJ, Stagliano KE, Deb D, Ellis MA, Carchman EH, Das A (2004). Tumor-derived p53 mutants induce oncogenesis by transactivating growth-promoting genes. Oncogene..

[56] Li H, Ning S, Ghandi M, Kryukov GV, Gopal S, Deik A (2019). The landscape of cancer cell line metabolism. Nat Med.

[57] Fenoglio-Preiser CM, Wang J, Stemmermann GN, Noffsinger A (2003). TP53 and gastric carcinoma: a review. Hum Mutat.

[58] Tornesello ML, Buonaguro L, Tatangelo F, Botti G, Izzo F, Buonaguro FM (2013). Mutations in TP53, CTNNB1 and PIK3CA genes in hepatocellular carcinoma associated with hepatitis B and hepatitis C virus infections. Genomics..

[59] LeBoeuf SE, Wu WL, Karakousi TR, Karadal B, Jackson SR, Davidson SM (2020). Activation of oxidative stress response in cancer generates a druggable dependency on exogenous non-essential amino acids. Cell Metab.

[60] Koppula P, Zhuang L, Gan B. Cystine transporter SLC7A11/xCT in cancer: ferroptosis, nutrient dependency, and cancer therapy. Protein Cell. 2020. 10.1007/s13238-020-00789-5. 10.1007/s13238-020-00789-5 PMC8310547 33000412

[61] Timmerman LA, Holton T, Yuneva M, Louie RJ, Padro M, Daemen A (2013). Glutamine sensitivity analysis identifies the xCT antiporter as a common triple-negative breast tumor therapeutic target. Cancer Cell.

[62] Romero R, Sayin VI, Davidson SM, Bauer MR, Singh SX, LeBoeuf SE (2017). Keap1 loss promotes Kras-driven lung cancer and results in dependence on glutaminolysis. Nat Med.

[63] Lehmann S, Bykov VJ, Ali D, Andren O, Cherif H, Tidefelt U (2012). Targeting p53 in vivo: a first-in-human study with p53-targeting compound APR-246 in refractory hematologic malignancies and prostate cancer. J Clin Oncol.

[64] Rudd SG, Tsesmetzis N, Sanjiv K, Paulin CB, Sandhow L, Kutzner J (2020). Ribonucleotide reductase inhibitors suppress SAMHD1 ara-CTPase activity enhancing cytarabine efficacy. EMBO Mol Med.

[65] Makela P, Zhang SM, Rudd SG (2021). Drug synergy scoring using minimal dose response matrices. BMC Res Notes.

[66] Barretina J, Caponigro G, Stransky N, Venkatesan K, Margolin AA, Kim S (2012). The Cancer Cell Line Encyclopedia enables predictive modelling of anticancer drug sensitivity. Nature..

[67] Rees MG, Seashore-Ludlow B, Cheah JH, Adams DJ, Price EV, Gill S (2016). Correlating chemical sensitivity and basal gene expression reveals mechanism of action. Nat Chem Biol.

[68] Ianevski A, Giri AK, Aittokallio T (2020). SynergyFinder 2.0: visual analytics of multi-drug combination synergies. Nucleic Acids Res.

