# Correction: Mutant p53-reactivating compound APR-246 synergizes with asparaginase in inducing growth suppression in acute lymphoblastic leukemia cells

**DOI:** 10.1038/s41419-022-05130-y

**Published:** 2022-08-03

**Authors:** Sophia Ceder, Sofi E. Eriksson, Ying Yu Liang, Emarndeena H. Cheteh, Si Min Zhang, Kenji M. Fujihara, Julie Bianchi, Vladimir J. N. Bykov, Lars Abrahmsen, Nicholas J. Clemons, Pär Nordlund, Sean G. Rudd, Klas G. Wiman

**Affiliations:** 1 Department of Oncology-Pathology, Karolinska Institutet, BioClinicum J6, Stockholm, Sweden; 2 Institute of Molecular and Cellular Biology, A*STAR, Singapore, Singapore; 3 Science For Life Laboratory, Department of Oncology-Pathology, Karolinska Institutet, Solna, Sweden; 4 Cancer Research Division, Peter MacCallum Cancer Centre, Melbourne, VIC Australia; 5 Sir Peter MacCallum Department of Oncology, The University of Melbourne, Parkville, VIC Australia; 6 Aprea Therapeutics AB, Solna, Sweden

**Keywords:** Cancer therapy, Haematological cancer

Correction to: *Cell Death & Disease* 10.1038/s41419-021-03988-y, published online 15 July 2021

The original version of this article was unfortunately missing additional information in the legend of figure 3. The following sentences should be added at the end of the legend to Fig. 3: The same GAPDH control blot is shown in panels F and J since both xCT (F) and ASNS (J) were examined on the same blot. The blot was divided into an upper part which was probed with ASNS antibody and a lower part which was probed with xCT antibody and then re-probed with GAPDH antibody. Thus, the same GAPDH blot serves as control for both xCT and ASNS. The legends to the Supplementary figures were also missing. The original article has been corrected.

