# Supplementary material for: Mutant p53-reactivating compound APR-246 synergizes with asparaginase in inducing growth suppression in acute lymphoblastic leukemia cells
Source: Cell Death Dis. 2021 Jul 15;12(7):709. doi: 10.1038/s41419-021-03988-y (PMC8282662; doi:10.1038/s41419-021-03988-y)
Supplement: Supplementary file 1 — Supplementary Tables [file 41419_2021_3988_MOESM1_ESM.docx]

**Table S1.** **Number of proteins identified by MS-CETSA**

| **Temperature:** | **37 °C**  Rep. 1 Rep. 2 | | **46 °C**  Rep. 1 Rep. 2 | | **52 °C**  Rep. 1 Rep. 2 | | **58 °C**  Rep. 1 Rep. 2 | |
| --- | --- | --- | --- | --- | --- | --- | --- | --- |
| **Number of detected proteins:** | 2958 | 3956 | 2515 | 3498 | 2477 | 2775 | 2555 | 2654 |
| **Number of proteins after clean-up:** | 2740 | 3697 | 2329 | 3217 | 2325 | 2431 | 2414 | 2374 |

###

**Table S2.** ***TP53* status and culturing of cell lines included in the study**

| **Cell line** | ***TP53* status*** | **Disease (cell type), age of patient** | **Culture media** | **Source** |
| --- | --- | --- | --- | --- |
| **CCRF-CEM** | p.R175H, p.R248Q | ALL (T cell), 4 years | RPMI-1640 media (Hyclone) supplemented with L-Glutamine and 10% FBS | Purchased from ATCC |
| **CCRF-SB** | WT | ALL (B cell), 11.5 years |  | Purchased from ATCC |
| **Jurkat** | p.R196*, p.T256A, p.D259G, p.S260A | ALL (T cell), 14 years |  | Katja Pokrovskaja |
| **Jurkat A3** | *Fas-selected subline of Jurkat* |  |  | Purchased from ATCC |
| **KARPAS 45** | p.R175H, p.R273C | ALL (T cell), 2 years | RPMI-1640 media containing Hepes (Hyclone) supplemented with L-Glutamine and 10% FBS | Purchased from Merck |
| **MOLT-4** | p.R306*, p.R248Q, WT | ALL (T cell), 19 years | RPMI-1640 media (Hyclone) supplemented with L-Glutamine and 10% FBS | Purchased from ATCC |
| **MOLT-16** | p.M237R, p.G244C, p.R158H | ALL (T cell), 5 years | IMDM media (Hyclone) supplemented with L-Glutamine and 10% FBS | Purchased from DSMZ |
| **OVCAR-3** | p.R248Q | Ovarian adenocarcinoma (epithelial), 60 years | RPMI-1640 media containing HEPES (Hyclone) supplemented with L-Glutamine, 10% FBS and insulin-transferrin-selenium (51300044, Thermo Fisher Scientific) | Aprea Therapeutics |
| **Reh** | WT, p.R181C | ALL (lymphoblast, non-T, non-B cell), | RPMI-1640 media (Hyclone) supplemented with L-Glutamine and 10% FBS | Purchased from ATCC |
| **RS4;11** | WT | ALL (lymphoblast), 32 years | RPMI-1640 media containing Hepes (Hyclone) supplemented with L-Glutamine and 10% FBS | Katja Pokrovskaja |
| **SUP-B15** | WT | ALL (B cell), 8 years |  | Katja Pokrovskaja |

** TP53 status as reported in the IARC or p53.fr database*

### Table S3. APR-246 IC_50_

| **Cell line** | **IC_50_ APR-246 (µM)** | **n** |
| --- | --- | --- |
| **CCRF-CEM** | 1.8 | 4 |
| **CCRF-SB** | 9.4 | 4 |
| **Jurkat** | 2.3 | 2 |
| **Jurkat A3** | 2.7 | 4 |
| **KARPAS-45** | 3.0 | 2 |
| **MOLT-4** | 2.9 | 4 |
| **MOLT-16** | 1.2 | 4 |
| **Reh** | 2.5 | 4 |
| **RS4;11** | 0.7 | 1 |
| **SupB15** | 2.5 | 1 |

### Table S4. Growth suppression ASNase treatment

|  |  | Growth suppression (%) | | | | | | | | | |
| --- | --- | --- | --- | --- | --- | --- | --- | --- | --- | --- | --- |
| ASNase (U/ml) |  | **CCRF-CEM** | **CCRF-SB** | **Jurkat** | **Jurkat A3** | **KARPAS-45** | **Molt-4** | **Molt-16** | **Reh** | **RS4;11** | **SupB15** |
| 0 | mean | -7,1 | -4,1 | -21,2 | -4,0 | 8,8 | -10,7 | -9,6 | -3,6 | -43,2 | 1,1 |
|  | SEM | 6,7 | 4,4 | 2,9 | 5,2 | 7,2 | 6,2 | 8,2 | 2,9 | 7,4 | 5,2 |
|  | n | 7 | 7 | 2 | 7 | 2 | 7 | 5 | 5 | 3 | 3 |
| 0.00002 | mean | -2,9 | 4,2 | -13,1 | -7,0 | 12,7 | -6,0 | -2,6 | -1,7 | 2,9 | 0,8 |
|  | SEM | 3,8 | 0,0 | 1,4 | 0,0 | 1,2 | 5,3 | 7,7 | 4,7 | 5,5 | 0,9 |
|  | n | 5 | 1 | 2 | 1 | 2 | 5 | 4 | 4 | 2 | 2 |
| 0.0024-0.0026 | mean | 15,3 | -17,3 | -5,3 | 11,3 | 13,2 | 21,5 | 27,1 | 0,2 | 41,0 | 53,6 |
|  | SEM | 8,0 | 9,6 | 2,0 | 12,3 | 3,0 | 6,4 | 13,8 | 2,8 | 37,5 | 29,6 |
|  | n | 6 | 2 | 2 | 2 | 2 | 6 | 4 | 4 | 2 | 2 |
| 0.003-0.0032 | mean | 49,4 | 0,2 | 63,4 | 11,8 | 57,3 | 40,7 | 51,9 | 26,6 | 95,3 | 91,7 |
|  | SEM | 3,6 | 3,1 | 1,8 | 2,9 | 2,4 | 4,0 | 3,0 | 3,0 | 0,8 | 2,1 |
|  | n | 6 | 2 | 2 | 2 | 2 | 6 | 4 | 4 | 2 | 2 |
| 0.04-0.2 | mean | 52,6 | -2,4 | 62,3 | 23,5 | 61,9 | 42,7 | 60,4 | 26,5 | 85,3 | 93,4 |
|  | SEM | 3,3 | 3,1 | 2,0 | 2,1 | 1,5 | 4,2 | 2,7 | 3,9 | 1,2 | 1,8 |
|  | n | 7 | 7 | 2 | 7 | 2 | 6 | 5 | 5 | 2 | 2 |
| 0.5-0.63 | mean | 91,8 | 69,5 | 97,5 | 93,5 | 95,1 | 92,1 | 99,7 | 78,4 | 83,1 | 98,8 |
|  | SEM | 0,9 | 0,0 | 0,2 | 0,0 | 0,1 | 1,3 | 0,8 | 2,4 | 0,0 | 0,0 |
|  | n | 5 | 1 | 2 | 1 | 2 | 5 | 5 | 5 | 1 | 1 |

*SEM = standard error of the mean*

### Table S5. Area-under-curve (AUC) based on cell viability of APR-246 with or without ASNase

|  | **AUC based on cell viability after 72h APR-246*** | | | | | | | | | | | |
| --- | --- | --- | --- | --- | --- | --- | --- | --- | --- | --- | --- | --- |
| **Cell line** | **ASNase (U/ml)** | **AUC** | **SEM** | **n** | **ASNase (U/ml)** | **AUC** | **SEM** | **n** | **ASNase (U/ml)** | **AUC** | **SEM** | **n** |
| **CCRF-CEM** | 0 | 223,8 | 29,9 | 5 | 0.04 | 99,14 | 23,69 | 4 | 0.5 | 37,44 | 10,59 | 4 |
| **CCRF-SB** | 0 | 484,4 | 81,6 | 5 | 0.05 | 476,4 | 44,95 | 4 | 0.4 | 144,8 | 32,29 | 4 |
| **Jurkat** | 0 | 300,6 | 25,6 | 2 | 0.04 | 93,56 | 6,70 | 2 | 0.5 | 24,78 | 0,85 | 2 |
| **Jurkat A3** | 0 | 289,0 | 80,7 | 5 | 0.05 | 180,3 | 12,25 | 4 | 0.4 | 48,74 | 6,74 | 4 |
| **KARPAS-45** | 0 | 369,6 | 10,3 | 2 | 0.04 | 200,0 | 5,73 | 2 | 0.5 | 89,41 | 14,56 | 2 |
| **MOLT-4** | 0 | 364,9 | 33,2 | 5 | 0.04 | 210,3 | 35,74 | 4 | 0.5 | 38,00 | 3,21 | 4 |
| **MOLT-16** | 0 | 185,4 | 31,4 | 5 | 0.04 | 58,38 | 18,05 | 4 | 0.5 | 16,25 | 16,01 | 4 |
| **Reh** | 0 | 290,3 | 27,7 | 5 | 0.04 | 240,4 | 29,71 | 4 | 0.5 | 128,0 | 18,35 | 4 |
| **RS4;11** | 0 | 284,3 | 34,8 | 2 | 0.0009 | 44,56 | 0,00 | 1 | 0.003 | 25,22 | 0,00 | 1 |
| **SupB15** | 0 | 284,1 | 8,0 | 2 | 0.0009 | 155,6 | 0,00 | 1 | 0.003 | 64,67 | 0,00 | 1 |

### **Area under the curve (AUC) was assessed over the concentration range of 0, 0.1, 0.3, 1, 3 and 10 μM APR-246 after 72h treatment as assessed by resazurin assay*

### Table S6. Synergy scores upon combination treatment ASNase +/- APR-246

|  | **ZIP Synergy Score^#^** | | **Bliss Synergy Score^#^** | | **HSA Synergy Score^#^** | |  |
| --- | --- | --- | --- | --- | --- | --- | --- |
| **Cell line** | **Mean** | **SEM** | **Mean** | **SEM** | **Mean** | **SEM** | **n** |
| **CCRF-CEM** | 7,1 | 3,0 | 6,3 | 2,7 | 11,5 | 3,6 | 4 |
| **CCRF-SB** | 8,7 | 1,2 | 8,8 | 1,4 | 13,2 | 1,5 | 4 |
| **Jurkat** | 9,6 | 2,3 | 4,8 | 2,5 | 7,0 | 2,0 | 2 |
| **Jurkat A3** | 18,1 | 2,7 | 17,5 | 2,5 | 18,0 | 3,2 | 4 |
| **KARPAS-45** | 4,1 | 3,0 | 2,9 | 2,0 | 6,3 | 0,7 | 2 |
| **MOLT-4** | 6,4 | 1,2 | 4,7 | 1,1 | 6,6 | 0,8 | 4 |
| **MOLT-16** | 6,9 | 1,9 | 6,7 | 1,7 | 10,8 | 1,4 | 4 |
| **Reh** | 3,5 | 0,7 | 2,2 | 0,3 | 3,5 | 1,7 | 4 |
| **RS4;11** | 16,0 | 0,0 | 15,9 | 0,0 | 19,0 | 0,0 | 1 |
| **SupB15** | 6,6 | 0,0 | 4,7 | 0,0 | 8,3 | 0,0 | 1 |

*^#^ Synergy score in most synergistic area*
