# Supplementary material for: Mutant p53-reactivating compound APR-246 synergizes with asparaginase in inducing growth suppression in acute lymphoblastic leukemia cells
Source: Cell Death Dis. 2021 Jul 15;12(7):709. doi: 10.1038/s41419-021-03988-y (PMC8282662; doi:10.1038/s41419-021-03988-y)
Supplement: Supplementary file 6 — Supplementary Figure Legends [file 41419_2021_3988_MOESM6_ESM.docx]

**Ceder et al. CDDIS-21-1734RR**

Mutant p53-reactivating compound APR-246 synergizes with asparaginase in inducing growth suppression in acute lymphoblastic leukemia cells

**Supplementary Figure legends**

**Figure S1. MS-CETSA identifies asparagine synthetase (ASNS) as a putative MQ target.**

A. Cell viability of OVCAR-3 cells determined by trypan blue exclusion of samples for MS-CETSA. The 50 and 200 μM MQ samples were excluded from MS-CETSA due to high cell death. Gradient indicates 0.0025, 0.01, 0.05, 0.2, 0.8, 3.1 and 12.5 μM MQ. n = 1.

B. Western blot of xCT, p53 (DO-1) and GAPDH in OVCAR-3 cells after 2 or 24 h treatment with indicated concentrations of MQ. Ponceau staining of the same membrane is shown to the right. n = 1

C. Glutathione reductase (80nM) activity after 40 or 80 min incubation with MQ at indicated concentrations. n = 1

D. Brightfield images of OVCAR-3 cells after indicated concentrations of MQ taken at indicated time points.

E. Cleaved caspase 3 activity (green cells normalized to confluence) determined by Incucyte over time. n =1.

F. Reaction catalyzed by ASNS.

**Figure S2. MQ thermostabilizes ASNS in ALL cells.**

A. Analysis of the DepMap portal of the Broad Institute of cancer cell lines ( > 300) showing PRIMA-1 activity area under the curve (AUC) grouped into ASNS protein expression high (above median) or low (below median).

B. Same WB-CETSA as shown in Fig. 2B with different exposure times and Ponceau staining.

C. Same quantification as in Fig. 2C but depicted as histogram. n = 1

**Figure S3. Markers for ASNase and APR-246 sensitivity in ALL cells.**

A. Same Western blot as shown in Fig. 3B but with different exposure time and strong bands covered. ASNS expression and Ponceau staining are also shown. n = 1

B. Western blot of untreated ALL cell lines showing thioredoxin reductase 1 (TrxR1) and GAPDH expression. Below is a heat map indicating growth suppression as determined by rezasurin measurement after 72h of APR-246 treatment. n = 1

C. Total glutathione (GSH + GSSG) determined by recycling assay in untreated ALL cell

16 lines after 24h in culture and area under curve (AUC) measured in viability after

72h of APR-246 treatment determined by resazurin assay. n = 1. Spearman correlation -0.02, p = 1.0 or excluding CCRF-SB r = 0.35, p = 0.4.

D. Total glutathione (GSH + GSSG) determined by recycling assay in untreated ALL cell lines after 24h or 72h in culture and IC_50_ values after 72h of APR-246 treatment determined by resazurin assay. n = 1. Spearman correlation at 24h r = 0.6, p = 0.1 or 72h r = 0.63, p = 0.8. CCRF-SB (IC_50_ > 9) has been excluded.

E. Analysis of the DepMap portal of the Broad Institute of 22 ALL cell lines of PRIMA-1 activity area under the curve (AUC) and GSH abundance. Spearman correlation r = 0.25 and p = 0.26.

F. Analysis of the DepMap portal of the Broad Institute of 22 ALL cell lines of PRIMA-1 activity AUC and asparagine abundance. Spearman correlation r = 0.28 and p = 0.21.

G. Analysis of the DepMap portal of the Broad Institute of 22 ALL cell lines of PRIMA-1 AUC and aspartate abundance. Spearman correlation r = -0.46 and *p = 0.03.

H. Analysis of the DepMap portal of the Broad Institute of PRIMA-1 activity AUC and asparagine abundance in 708 cancer cell lines of various origin. Spearman correlation r = 0.045 and p = 0.23.

I. Analysis of the DepMap portal of the Broad Institute of PRIMA-1 activity AUC and aspartate abundance in 708 cancer cell lines of various origin. Spearman correlation r =

-0.06 and p = 0.11.

**Figure S4. APR-246 synergizes with ASNase in ALL cells.**

A. Cell viability determined by resazurin assay in ALL cell lines after 72h of increasing concentrations of ASNase (three sets of concentrations ranges: low, mid and high) in combination with APR-246. Each dot indicates an individual experiment

B. Heat map of MOLT-16, Jurkat A3 and CCRF-CEM cells indicating average growth suppression determined by resazurin assay after 72h treatment with APR-246 (0.1, 0.3, 1, 3 and 10 μM) and ASNase (0.00002, 0.0003, 0.003, 0.04 and 0.5 U/ml for Molt-16 and CCRF-CEM and 0.02, 0.05, 0.1, 0.4 and 1 U/ml for Jurkat A3). n = 4-5.

C. Correlation synergy score of most synergistic area determined by ZIP model and quantification of p53 normalized to GAPDH expression by Western blot (from Fig. 3) in 10 ALL cell lines. Pearson r = -0.37 and p = 0.3.

D. Correlation synergy score of most synergistic area determined by ZIP model and quantification of xCT normalized to GAPDH expression by Western blot (from Fig. 3) in

10 ALL cell lines. Pearson r = -0.24 and p = 0.5.

E. Correlation synergy score of most synergistic area determined by ZIP model and quantification of ASNS normalized to GAPDH expression by Western blot (from Fig. 3) in 10 ALL cell lines. Pearson r = -0.33 and p = 0.4.

F. Violin plot of ASNS protein expression grouped by TP53 status in the TCGA PanCancer atlas studies (32 studies). *p < 0.0001, Kurskal-Wallis test and Dunn’s multiple comparisons test.
